# Effects of capsular tension ring on surgical outcomes of premium intraocular lens in patients with suspected zonular weakness

**DOI:** 10.1371/journal.pone.0228999

**Published:** 2020-02-24

**Authors:** Teruyuki Miyoshi, Shuhei Fujie, Hironori Yoshida, Hiroshi Iwamoto, Hideki Tsukamoto, Tetsuro Oshika

**Affiliations:** 1 Miyoshi Eye Clinic, Hiroshima, Japan; 2 Department of Ophthalmology, Faculty of Medicine, University of Tsukuba, Tsukuba, Ibaraki, Japan; National Taiwan University Hospital, TAIWAN

## Abstract

**Purpose:**

To assess the influence of capsular tension ring (CTR) on surgical outcomes of toric and multifocal intraocular lenses (IOLs) in eyes at high risk of zonular instability.

**Methods:**

Fifty-five eyes of 43 patients who had undergone phacoemulsification and IOL implantation were included in the analysis. They had some risk of zonular weakness, such as pseudoexfoliation, shallow anterior chamber, high myopia, and phacodonesis, or were judged to have unstable zonules during surgery. Toric IOL was implanted in 9 eyes with CTR and 22 eyes without CTR, while multifocal IOL was used in 9 eyes with CTR and 15 eyes without CTR. Manifest refraction, refractive astigmatism, visual acuity, and degree of IOL decentration and tilt measured using swept-source anterior segment optical coherence tomography were analyzed. Axis misalignment of toric IOLs were also evaluated.

**Results:**

In toric IOLs, co-implantation of CTR significantly reduced decentration and axis misalignment of IOL, resulting in better uncorrected and corrected visual acuity after surgery. In multifocal IOLs, combined use of CTR significantly prevented IOL tilt, leading to better intermediate visual acuity. Spherical equivalent and residual astigmatism were not significantly affected by the use of CTR.

**Conclusions:**

CTR reduces decentration and axis misalignment of toric IOL and tilt of multifocal IOL, achieving improvement of postoperative visual function in eyes with suspected zonular instability.

## Introduction

Cataract surgery in eyes with compromised zonular integrity can be quite challenging, with increased risks of vitreous prolapse, capsular rupture, retained lens material, and postoperative intraocular lens (IOL) dislocation. Malposition of IOL can induce significant symptoms such as refractive error, anisometropia, and marked visual disturbances. Capsular tension ring (CTR) was developed to provide both intraoperative and postoperative stabilization of the capsular bag and IOL in the setting of zonular weakness or loss [1]. Many studies demonstrated that CTR can prevent marked IOL decentration, tilt, and severe anterior capsule contraction, and may lead to prevention of refractive prediction error in eyes with zonular dehiscence or weakness [2–4].

Optimal positioning and centration of IOL are important in all pseudophakic eyes, but especially so in cases with premium IOLs such as toric and multifocal lenses. Several studies have reported that co-implantation of CTR and toric IOL was a safe and effective technique for ensuring better rotational stability of toric IOLs [5–7]. The combined use of CTR and multifocal IOL has also been evaluated in terms of refractive predictability and stability [8–11]. These studies, however, were carried out in cases with standard cataract surgery, and there has been no study which assessed combined use of CTR and premium IOLs in eyes at high risk of zonular instability. The current study was aimed at investigating the effects of CTR on the surgical outcomes of toric and multifocal IOLs in eyes with suspected zonular weakness.

## Patients and methods

### Patients

In this retrospective study, 55 eyes of 43 patients (70.7 ± 10.3 years old, mean ± standard deviation) who had undergone phacoemulsification and implantation of toric or multifocal IOL from January 2015 to December 2018 were recruited for postoperative examinations. They had some risk of zonular weakness, such as pseudoexfoliation, shallow anterior chamber, high myopia, and phacodonesis, or were judged to have compromised zonular integrity during surgery. None of the eyes had any history of previous ocular surgery except for cataract removal. Eyes were not included if they had any ocular diseases which can affect surgical outcomes. Eyes were excluded if there were any intraoperative complications which can affect IOL stability, except for zonular instability. In cases of severe zonular dehiscence, the use of premium IOL was aborted and such cases were not included in the current analysis. Postoperatively, the patients were followed up for at least 3 months. All patients provided a written informed consent. The study protocol was approved by the institutional review board of Miyoshi Eye Clinic, and the study adhered to the tenets of the Declaration of Helsinki.

### Intraocular lenses and surgery

Toric IOL (TECNIS Toric ZCT, Johnson & Johnson Vision, Inc., Santa Ana, CA) and multifocal IOL (TECNIS Multifocal ZLB00, Johnson & Johnson Vision, Inc.) were used. Standard CTR (CTR130A0, HOYA, Tokyo, Japan) was inserted into the capsular bag during surgery according to intraoperative judgement of the surgeon. We did not use modified CTR and capsular tension segment with fixation eyelets to anchor the capsular bag to the scleral wall.

One surgeon (TM) performed all cases using a standard technique of phacoemulsification through a 2.4-mm temporal clear corneal incision. Anterior capsulorhexis of approximately 5.0 mm in diameter was created and the IOL was implanted into the capsular bag with an injector. It was confirmed by direct observation that all IOLs were implanted within the intact continuous capsulorhexis and IOL optic was completely covered by the capsulorhexis edge. Sutures were not used to close the incision.

### Examinations

Manifest refraction, preoperative corneal astigmatism, postoperative refractive astigmatism, and uncorrected (UDVA) and corrected distance visual acuity (CDVA) were measured. In eyes with multifocal IOLs, postoperative uncorrected intermediate visual acuity (UIVA) at 70 cm, and uncorrected near visual acuity (UNVA) at 40 and 30 cm were measured.

The degree of IOL decentration and tilt was measured using swept-source anterior segment optical coherence tomography (AS-OCT, CASIA2, Tomey Corp., Nagoya, Japan) [12,13]. This instrument performs a three-dimensional analysis with 16 different angles of AS-OCT images, then automatically measures the amount of IOL decentration and tilt relative to the visual axis. The degree of IOL decentration is presented as the absolute value (mm) relative to the visual axis. The degree of IOL tilt is shown as the absolute value (degree) relative to the visual axis. The alignment of toric IOL axis was evaluated on high-resolution, slit-lamp digital retroillumination photographs taken with a dilated pupil. The postoperative and preoperative images were compared to assess the degree of IOL axis misalignment, during which two images were aligned using iris or scleral landmarks to eliminate the influence of head tilt, ocular cyclotorsion, or other alignment anomalies. The examiners and patients were blinded to the allocation of the eye to either group.

### Statistical analysis

Numerical data were compared between groups using the Wilcoxon signed-rank test. Statistical analysis was performed using SPSS Statistics for Windows software (version 25, IBM Corp., Armonk, NY, USA). In all cases, the level of significance was a p-value less than 0.05.

## Results

### Background data

Preoperative characteristics of eyes with toric and multifocal IOLs are shown in Tables 1 and 2, respectively. In eyes with toric IOL, there was no significant difference in the baseline characteristics between the CTR and no-CTR groups. In cases treated with multifocal IOL, the follow-up period was longer in eyes without CTR than in those with CTR, but other parameters were compatible between groups. Toric IOL was implanted in 9 eyes with CTR and 22 eyes without CTR, while multifocal IOL was used in 9 eyes with CTR and 15 eyes without CTR. Risk factors of zonular weakness are summarized in Table 3.

**Table 1:** Preoperative characteristics of eyes in the toric IOL group.

	With CTR	Without CTR	p-value
Male/female (eyes)	2/7	14/8	
Age (years)	70.3 ± 14.1	77.7 ± 6.7	0.124
Follow up (months)	15.6 ± 10.9	19.4 ± 10.9	0.507
Corneal astigmatism (D)	1.79 ± 0.44	1.83 ± 0.36	0.881
Axial length (mm)	24.3 ± 1.5	23.3 ± 0.9	0.086
Target refraction (D)	-0.14 ± 0.16	-0.12 ± 0.13	0.881
Anterior chamber depth (mm)	3.15 ± 0.43	3.24 ± 0.28	0.814

IOL = intraocular lens, CTR = capsular tension ring, D = diopter

**Table 2:** Preoperative characteristics of eyes in the multifocal IOL group.

	With CTR	Without CTR	p-value
Male/female (eyes)	4/5	0/15	
Age (years)	66.3 ± 6.7	63.4 ± 7.4	0.640
Follow up (months)	12.7 ± 9.8	24.2 ± 5.7	0.002
Corneal astigmatism (D)	0.54 ± 0.25	0.81 ± 0.64	0.290
Axial length (mm)	25.1 ± 1.8	24.2 ± 1.2	0.215
Target refraction (D)	-0.13 ± 0.10	-0.13 ± 0.14	0.770
Anterior chamber depth (mm)	3.48 ± 0.17	3.35 ± 0.37	0.238

IOL = intraocular lens, CTR = capsular tension ring, D = diopter

**Table 3:** Risk factors of zonular weakness.

	Toric IOLs		Multifocal IOLs	
	With CTR	Without CTR	With CTR	Without CTR
Pseudoexfoliation	1	3	-	-
Phacodonesis	1	-	-	-
Shallow anterior chamber	2	3	-	2
High myopia	4	5	3	1
Advanced age	1	6	2	5
Unknown (intraoperative judgement of unstable zonules)	-	5	4	7

IOL = intraocular lens, CTR = capsular tension ring

### Toric IOL

Postoperative data of patients with toric IOL are summarized in Table 4. The eyes with CTR showed significantly better UDVA (p = 0.03) and CDVA (p = 0.018) than those without CTR. Postoperative spherical equivalent and residual astigmatism were not different between groups. The amounts of IOL decentration in eyes with CTR was significantly smaller than those without CTR (p = 0.037). The degree of IOL tilt was also smaller in the CTR group than the no-CTR group, though the statistical significance was marginal (p = 0.094). The amount of toric IOL axis misalignment was significantly smaller in eyes with CTR than without CTR (p = 0.037).

**Table 4:** Postoperative data of patients with toric IOLs.

	With CTR	Without CTR	p-value
Spherical equivalent (D)	0.00 ± 0.13	-0.09 ± 0.24	0.292
Refractive astigmatism (D)	0.11 ± 0.22	0.24 ± 0.43	0.623
Uncorrected distance visual acuity (logMAR)	-0.10 ± 0.08	0.00 ± 0.04	0.03
Corrected distance visual acuity (logMAR)	-0.10 ± 0.08	-0.02 ± 0.03	0.018
Decentration (mm)	0.23 ± 0.14	0.31 ± 0.12	0.037
Tilt (degree)	3.28 ± 1.14	3.46 ± 1.10	0.094
Axis misalignment (degree)	1.56 ± 1.01	2.44 ± 1.42	0.037

IOL = intraocular lens, CTR = capsular tension ring, D = diopter, logMAR = logarithm of minimum angle of resolution

### Multifocal IOL

**Fig 1:**
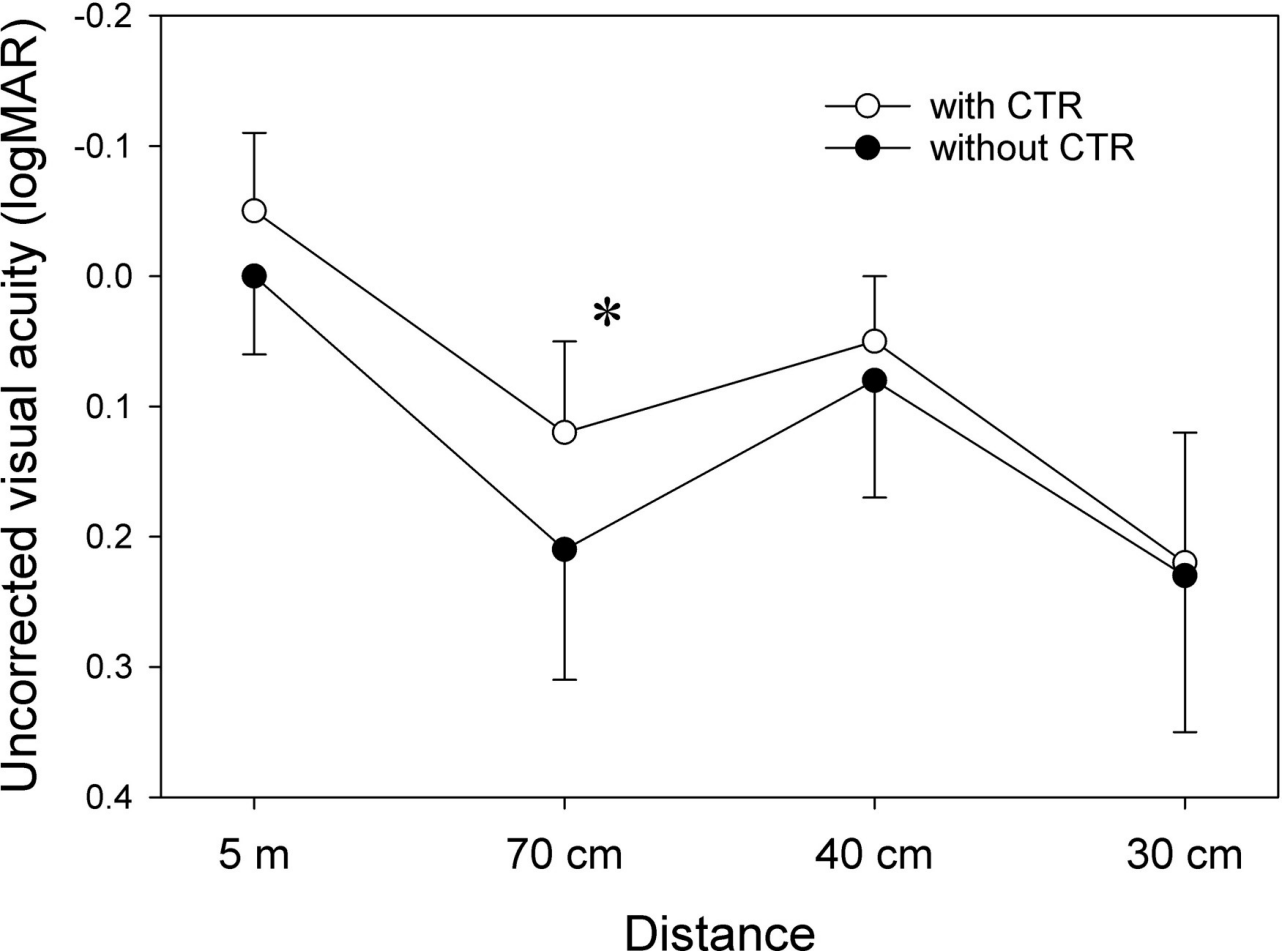
Uncorrected visual acuity in eyes with multifocal intraocular lenses. *p<0.05 (Wilcoxon signed-rank test).

Measurement data in patients treated with multifocal IOL are presented in Table 5. UIVA at 70 cm was significantly better in eyes with CTR than without CTR (p = 0.021). UDVA and UNVA tended to be better in eyes with CTR than without CTR (Fig 1), but the difference did not reach statistical significance. Postoperative refractive error and residual astigmatism were not different between groups. The degree of tilt was significantly smaller in the CTR group than in the no-CTR group (p = 0.025).

**Table 5:** Postoperative data of patients with multifocal IOLs.

	With CTR	Without CTR	p-value
Spherical equivalent (D)	-0.14± 0.22	-0.11 ± 0.28	0.861
Refractive astigmatism (D)	0.17 ± 0.28	0.30 ± 0.46	0.640
Uncorrected distance visual acuity (logMAR)	-0.05 ± 0.06	0.00 ± 0.06	0.138
Corrected distance visual acuity (logMAR)	-0.07 ± 0.07	-0.04 ± 0.05	0.318
Uncorrected intermediate visual acuity at 70 cm (logMAR)	0.12 ± 0.07	0.21 ± 0.10	0.021
Uncorrected near visual acuity at 40 cm (logMAR)	0.05 ± 0.05	0.08 ± 0.09	0.599
Uncorrected near visual acuity at 30 cm (logMAR)	0.22 ± 0.10	0.23 ± 0.12	0.861
Decentration (mm)	0.25 ± 0.27	0.19 ± 0.15	0.953
Tilt (degree)	2.90 ± 0.80	3.97 ± 1.45	0.025

IOL = intraocular lens, CTR = capsular tension ring, D = diopter, logMAR = logarithm of minimum angle of resolution

## Discussion

As shown in Table 3, patients’ background conditions are different between the toric and multifocal IOL groups. Eyes with higher risk of zonular weakness, such as pseudoexfoliation and phacodonesis, were included in the toric IOL group, while those eyes were precluded from the multifocal IOL group. It is a general consensus that pseudoexfoliation and phacodonesis are among the relative contraindications of multifocal IOLs [14]. Such different selection criteria should be responsible for the different results obtained in the toric and multifocal IOL groups.

In eyes that received toric IOLs, both UDVA and CDVA were significantly better in eyes with CTR than those without CTR, while postoperative spherical equivalent and residual astigmatism were not different between groups. The amounts of IOL decentration and axis misalignment were significantly smaller in eyes with CTR than those without CTR. The degree of IOL tilt was also smaller in the CTR group than the no-CTR group, though the statistical significance remained marginal. These results indicate that CTR improves surgical outcomes of toric IOL not only by reducing postoperative IOL rotation but also by ensuring better centration of IOL in eyes at higher risk of zonular instability. It has been reported that IOL decentration and tilt increase wavefront aberrations and degrade optical performance of the eye after cataract surgery [15–18]. Rastogi et al. conducted a prospective randomized clinical trial in standard cataract cases and reported that mean rotation of toric IOL at 3 months postoperatively was remarkably smaller in eyes with CTR (1.85 ±1.72 degrees) than in those without CTR (4.02 ± 2.04 degrees), with a statistically significant difference between groups [6]. Zhao et al. demonstrated that, in eyes with high axial myopia and cataract, CTR could effectively increase the rotational stability of toric IOLs, achieving improvement in corneal astigmatism and visual acuity [7]. Sagiv et al. reported a case of highly myopic eye in which 2 CTRs were required to fixate a toric IOL in the correct position during realignment surgery [5]. The rationale for implanting a CTR to improve toric IOL stability is that it theoretically enforces symmetry of the capsular bag, stretching the bag’s equator and thus flattening the bag in the anterior-posterior axis. The CTR may also increase the friction on the IOL haptics and thus enhance stability. The findings of this study indicate that improved centration and reduced tilt of the IOL also contribute to the better surgical outcomes of toric IOLs in eyes with suspected zonule instability.

In eyes treated with multifocal IOL, UIVA was significantly better in eyes with CTR than in those without CTR. UDVA was also better in eyes that received co-implantation of multifocal IOL and CTR, though the statistical significance was marginal. It seems that the difference would reach a significant level if the number of eyes enrolled in the study is increased. Postoperative refractive error and residual astigmatism were not different between groups, but the degree of tilt was significantly smaller in eyes with CTR than in those without CTR. These results indicate that better positioning of the IOL resulted in improved visual performance of multifocal IOLs after surgery. Mastropasqua et al. demonstrated that implantation of CTR with multifocal IOL reduced the ocular wavefront error related to a reduction of third-order aberration associated with better IOL position [10]. Nistad et al. reported that using CTR with trifocal IOL had no statistically significant effect on refractive stability [11]. Alió et al. indicated that combined use of CTR and plate haptic bifocal IOLs provided better IOL stability, resulting in good efficacy, predictability, and safety and increased intraocular optical performance after surgery [8,9]. These previous and present findings suggest that placement of CTR helps better positioning of multifocal IOL and improves visual outcomes after surgery, and such effects are more prominent in eyes at higher risk of IOL malpositioning.

When comparing the toric and multifocal IOL groups, the obtained results showed several discrepancies. The co-implantation of CTR and IOL reduced tilt in eyes with multifocal IOL but not in those with toric IOL. The CTR, however, did decrease tilt of toric IOL from 3.46 ± 1.10 degrees to 3.28 ± 1.14 degrees, with a marginal statistical difference (p = 0.094). With a larger study population, this difference would become significant. On the other hand, the use of CTR significantly prevented IOL decentration and tilt in eyes with toric IOL, but such tendency was not found in eyes with multifocal IOL. This seems to be due to the different selection criteria for toric and multifocal IOLs, with eyes having greater risk of zonular instability precluded from multifocal IOL implantation.

The current study has several limitations. First, this was a retrospective study, and random assignment of eyes to either CTR or no-CTR group was not conducted. The use of CTR was determined depending on the intraoperative judgement of the surgeon. Using such strategies, however, it seems natural to assume that the severity of zonular weakness tends to be greater in the CTR group than the no-CTR group. Nevertheless, the current findings demonstrated that the CTR group showed better postoperative performance than the no-CTR group, indicating the efficacy of CTR in eyes at high risk of zonular instability. Second, the follow-up period was significantly longer in the multifocal IOL without CTR group than the multifocal IOL with CTR group. This difference might have caused underestimation of IOL tilt in the CTR group if IOL tilt is progressive over time. At present, we have no clear answer for this and thus longer-term observation is necessary. Third, measurements of wavefront aberration were not conducted in this study, which might have shed more lights on the association between IOL positioning and visual performance of patients with premium IOL. Fourth, we did not evaluate multifocal toric IOLs. Such model was not available in our market at the time of current study. Future studies are awaited.

In conclusion, we retrospectively assessed the effects of CTRs on surgical outcomes of premium IOLs in eyes with suspected zonular instability. In toric IOLs, co-implantation of CTR significantly reduced decentration and toric axis misalignment, resulting in better uncorrected and corrected visual acuity after surgery. In multifocal IOLs, combined use of CTR significantly prevented IOL tilt, leading to better uncorrected visual acuity. CTR is a useful device to improve surgical outcomes of premium IOL in eyes at high risk of compromised zonular integrity.

## Supporting information

S1 Datasets (PDF)
