# Supplementary material for: Effects of capsular tension ring on surgical outcomes of premium intraocular lens in patients with suspected zonular weakness
Source: PLoS One. 2020 Feb 24;15(2):e0228999. doi: 10.1371/journal.pone.0228999 (PMC7039513; doi:10.1371/journal.pone.0228999)
Supplement: S1 Datasets — (PDF) [file pone.0228999.s001.pdf]

Effects of capsular tension ring on surgical outcomes of premium intraocular lens in patients with suspected zonular weakness submitted with DOI https://doi.org/10.5061/dryad.tmpg4f4vb. There may be a delay for processing before the item is available.

My Datasets

Start New Dataset

In Progress

You do not have any datasets in progress.

Submitted

Title	Status	Publication date	Actions
Effects of capsular tension ring on surgical outcomes of premium intraocular lens in patients with suspected zonular weakness	In Progress		Preview | Update

Copyright (c) 2019 Dryad
